# Supplementary material for: Spiro-containing derivatives show antiparasitic activity against Trypanosoma brucei through inhibition of the trypanothione reductase enzyme
Source: PLoS Negl Trop Dis. 2020 May 21;14(5):e0008339. doi: 10.1371/journal.pntd.0008339 (PMC7269337; doi:10.1371/journal.pntd.0008339)
Supplement: S1 Table — (PDF) [file pntd.0008339.s001.pdf]

Supporting Information

S1 Table Biological data for hits resulted from HTS and selected follow-up compounds

ID	Structure	TbTR assay IC$_{50}$ (nM) n=4	DTNB assay IC$_{50}$ (nM) n=4	hGSH assay IC$_{50}$ (nM) n=2	MS $[M+H]^+$ Found	[a]Purity (%)
**1**	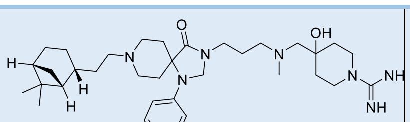	3501 ± 2187	966 ± 68	> 50000	608	95
**2**	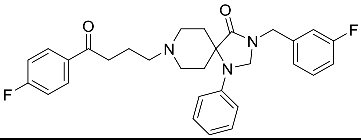	20351 ± 4888	20364 ± 3098	> 50000	504	99
**3**	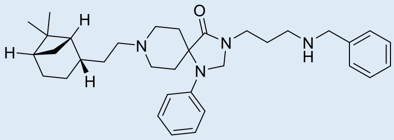	23270 ± 4884	16644 ± 3219	> 50000	529	95
**4**	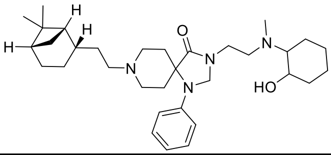	34098 ± 4850	27337 ± 7866	> 50000	537	99
**5**	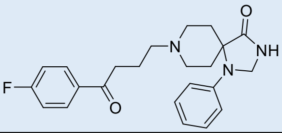	> 85000	--	--	395	99
**6**	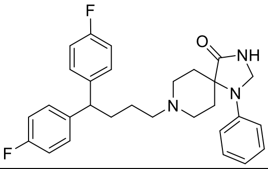	> 85000	--	--	476	99
**7**	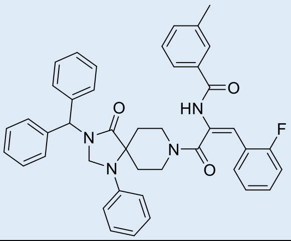	> 85000	--		679	90
**8**	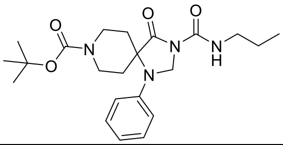	> 85000	--	--	417	99
**9**	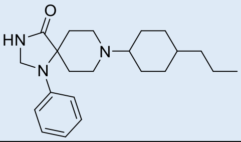	> 85000	--	--	356	98
**10**	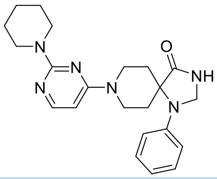	> 85000	--	--	393	98

Supporting Information

ID	Structure	TbTR assay IC_{50} (nM) n=4	DTNB assay IC_{50} (nM) n=4	hGSH assay IC_{50} (nM) n=2	MS $[M+H]^+$ Found	[a]Purity (%)
**11**	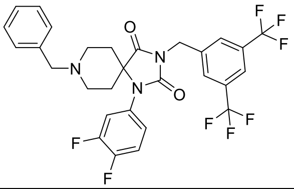	> 85000	--	--	598	99
**12**	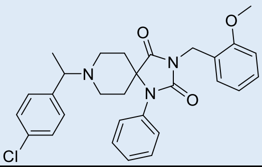	> 85000	--	--	505	94
**13**	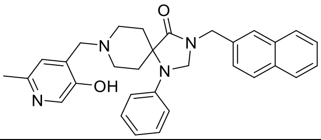	> 85000	--	--	493	99
**14**	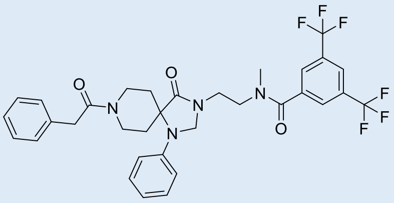	> 85000	--	--	647	91
**15**	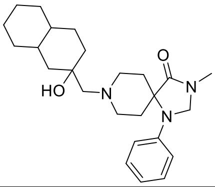	> 85000	--	--	412	99
**16**	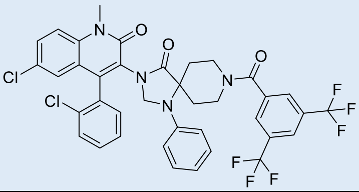	> 85000	--	--	774	99
**17**	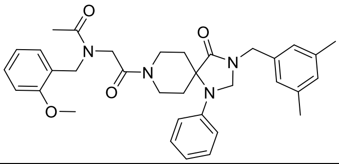	> 85000	--	--	569	99
**18**	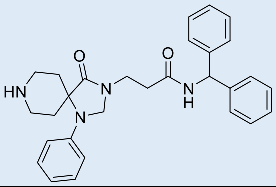	> 85000	--	--	469	95
**19**	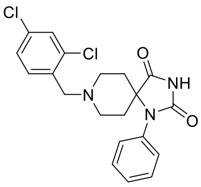	> 85000	--	--	405	99

Supporting Information

ID	Structure	TbTR assay IC$_{50}$ (nM) n=4	DTNB assay IC$_{50}$ (nM) n=4	hGSH assay IC$_{50}$ (nM) n=2	MS $[M+H]^+$ Found	[a]Purity (%)
**20**	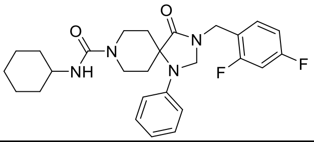	> 85000	--	--	483	99
**21**	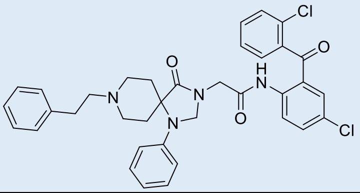	> 85000	--	--	642	99
**22**	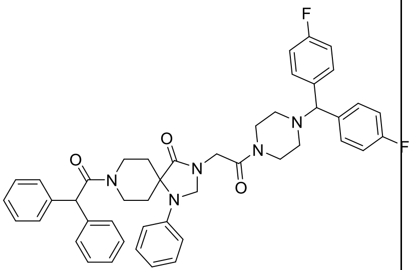	> 85000	--	--	754	92
**23**	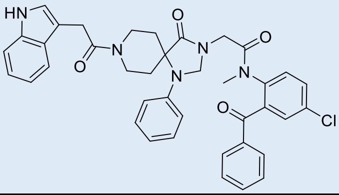	> 85000	--	--	675	99
**24**	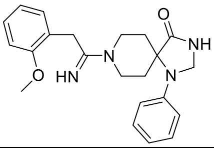	> 85000	--	--	379	99
**25**	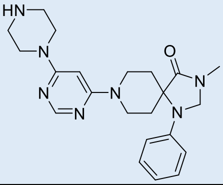	> 85000	--	--	407	95
**26**	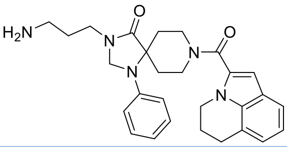	> 85000	--	--	472	90

[a]Purity was assessed by UPLC/MS using UV detection (diode array) as described in the experimental section of the main manuscript.
