# Supplementary material for: Spiro-containing derivatives show antiparasitic activity against Trypanosoma brucei through inhibition of the trypanothione reductase enzyme
Source: PLoS Negl Trop Dis. 2020 May 21;14(5):e0008339. doi: 10.1371/journal.pntd.0008339 (PMC7269337; doi:10.1371/journal.pntd.0008339)
Supplement: S2 Table — (PDF) [file pntd.0008339.s002.pdf]

Supporting Information

S2 Table. Crystal parameters, data collection statistics and refinement statistics of 1-TR complex.

**Crystal parameters**	
PDB code	6RB5
Space group	$P2_12_12_1$
Unit cell dimension (a,b,c) (Å)	63.1, 132.6, 161.0
Asymmetric unit composition (molecule, amount, modelled residue range)	TbTR, dimer (A:1-489, B:1-488)<br>FAD, 2<br>Compound 1, 4<br>Sulfate ions, 9<br>Glycerol, 6<br>H_2O, 486
Wilson B-factor (Å^2)	31.5
**Data analysis statistics**	
Resolution range (highest resolution shell) (Å)	102.39-1.98 (2.01-1.98)
Unique reflections	94907 (4693)
Completeness (%)	99.7 (99.9)
Redundancy	5.5 (5.4)
Rmerge (%)	7.1 (78.6)
CC(1/2) (%)	99.9 (78.8)
$\langle I/\sigma(I)\rangle$	13.3 (1.1)
**Refinement statistics**	
Resolution range (highest resolution bin)	102.39-1.98 (2.03-1.98)
R_{crys} (%)	18.3 (28.1)
R_{free} (%)	20.6 (28.7)
rms (angles) (°)	1.533
rms (bonds) (Å)	0.009
Mean B value (Å^2)	37.55
Residues in allowed region of Ramachandran plot / generously allowed (%)	100 (2)
