# Supplementary material for: Spiro-containing derivatives show antiparasitic activity against Trypanosoma brucei through inhibition of the trypanothione reductase enzyme
Source: PLoS Negl Trop Dis. 2020 May 21;14(5):e0008339. doi: 10.1371/journal.pntd.0008339 (PMC7269337; doi:10.1371/journal.pntd.0008339)
Supplement: S1 Fig — (PDF) [file pntd.0008339.s003.pdf]

Supporting Information

S1 Fig. ^{1}H NMR spectrum for compound **1**^{1}H NMR spectrum for compound **1.**

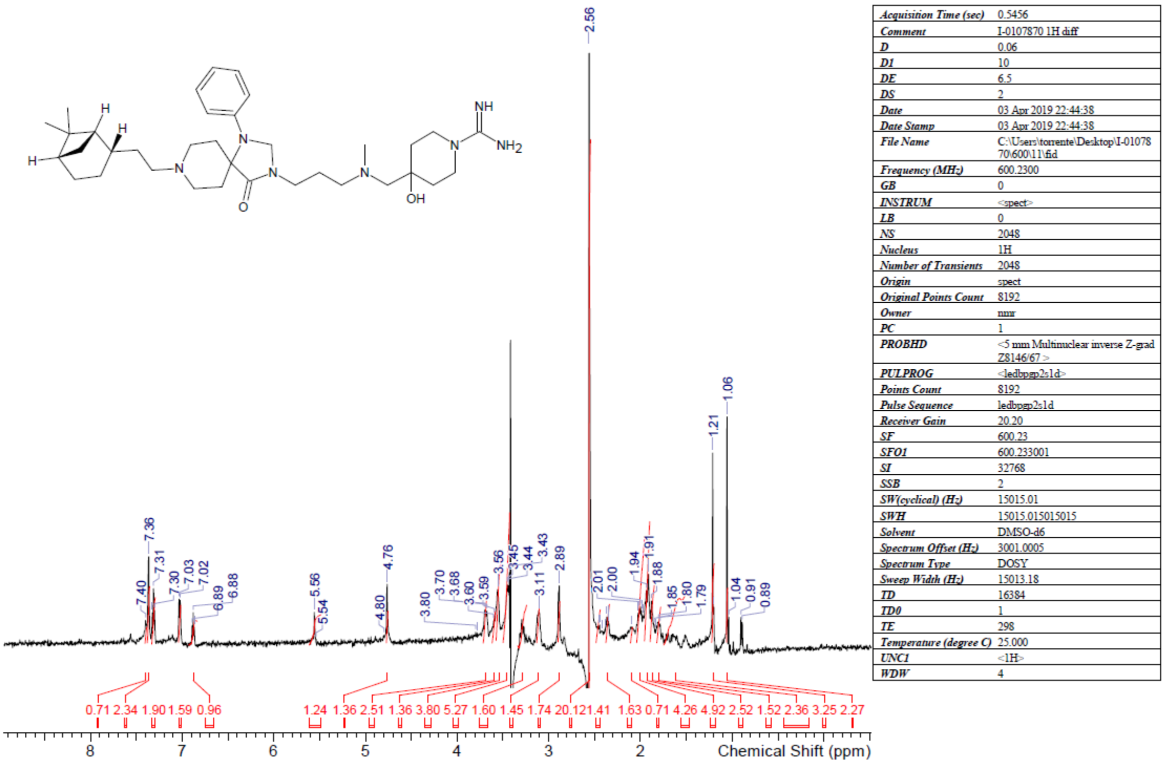

Acquisition Time (sec)	0.5456
Comment	I-0107870 1H diff
D	0.06
D1	10
DE	6.5
DS	2
Date	03 Apr 2019 22:44:38
Date Stamp	03 Apr 2019 22:44:38
File Name	C:\Users\torrente\Desktop\I-0107870\600\11\fid
Frequency (MHz)	600.2300
GB	0
INSTRUM	<spect>
LB	0
NS	2048
Nucleus	1H
Number of Transients	2048
Origin	spect
Original Points Count	8192
Owner	nmr
PC	1
PROBHD	<5 mm Multinuclear inverse Z-grad Z8146/67 >
PULPROG	<ledbpgp2s1d>
Points Count	8192
Pulse Sequence	ledbpgp2s1d
Receiver Gain	20.20
SF	600.23
SFO1	600.233001
SI	32768
SSB	2
SW(cyclical) (Hz)	15015.01
SWH	15015.015015015
Solvent	DMSO-d6
Spectrum Offset (Hz)	3001.0005
Spectrum Type	DOSY
Sweep Width (Hz)	15013.18
TD	16384
TD0	1
TE	298
Temperature (degree C)	25.000
UNC1	<1H>
WDW	4
