# Supplementary figures and images for: Spiro-containing derivatives show antiparasitic activity against Trypanosoma brucei through inhibition of the trypanothione reductase enzyme
Source: PLoS Negl Trop Dis. 2020 May 21;14(5):e0008339. doi: 10.1371/journal.pntd.0008339 (PMC7269337; doi:10.1371/journal.pntd.0008339)

## Supporting Information

**S2 Fig.** Electron density map of compound 1 bound to site 1 (A) or site 2 (B).

A

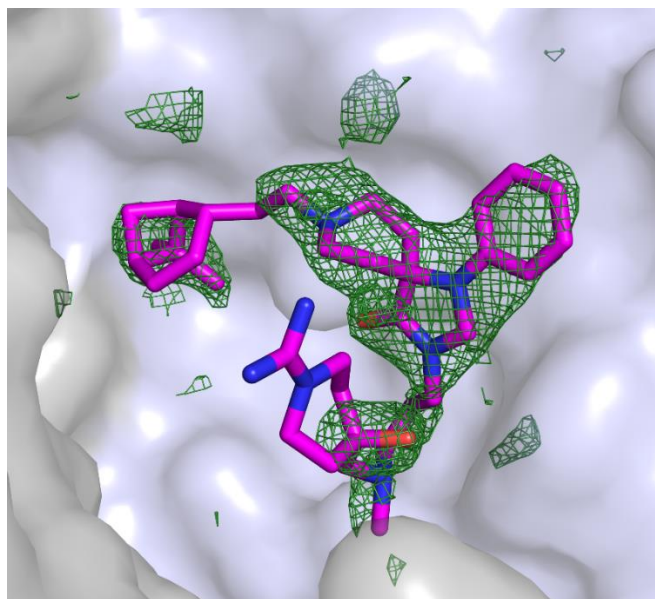

B

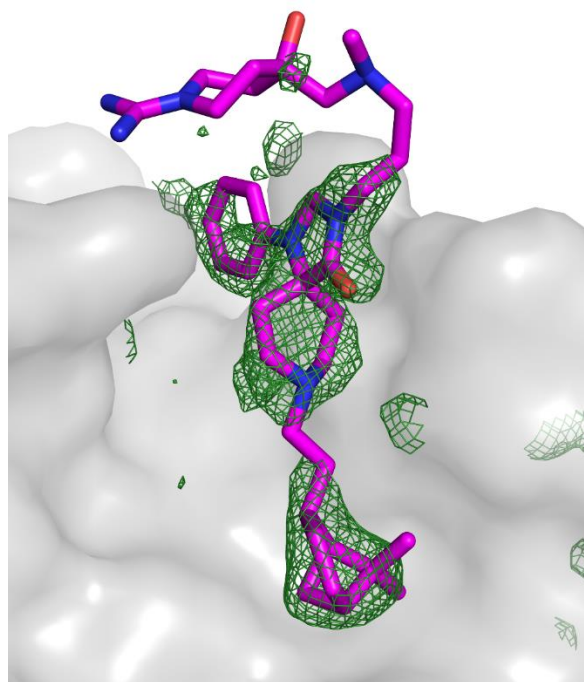

Supplement: S2 Fig — Electron density map of compound 1 bound to site 1 (A) or site 2 (B). (PDF) [file pntd.0008339.s004.pdf]
