# Supplementary material for: Spiro-containing derivatives show antiparasitic activity against Trypanosoma brucei through inhibition of the trypanothione reductase enzyme
Source: PLoS Negl Trop Dis. 2020 May 21;14(5):e0008339. doi: 10.1371/journal.pntd.0008339 (PMC7269337; doi:10.1371/journal.pntd.0008339)

Supporting Information

S3 Fig. The distinctly positive electrostatic potential of the GR substrate binding cavity is unsuitable for accommodating compound 1 due to the presence of positively charged carboximidamide arm and tertiary ammino group (green lateral chains) whereas the *Tb*TR cavity surface electrostatic potential (grey lateral chains) appears to be compatible with the binding of compound 1.

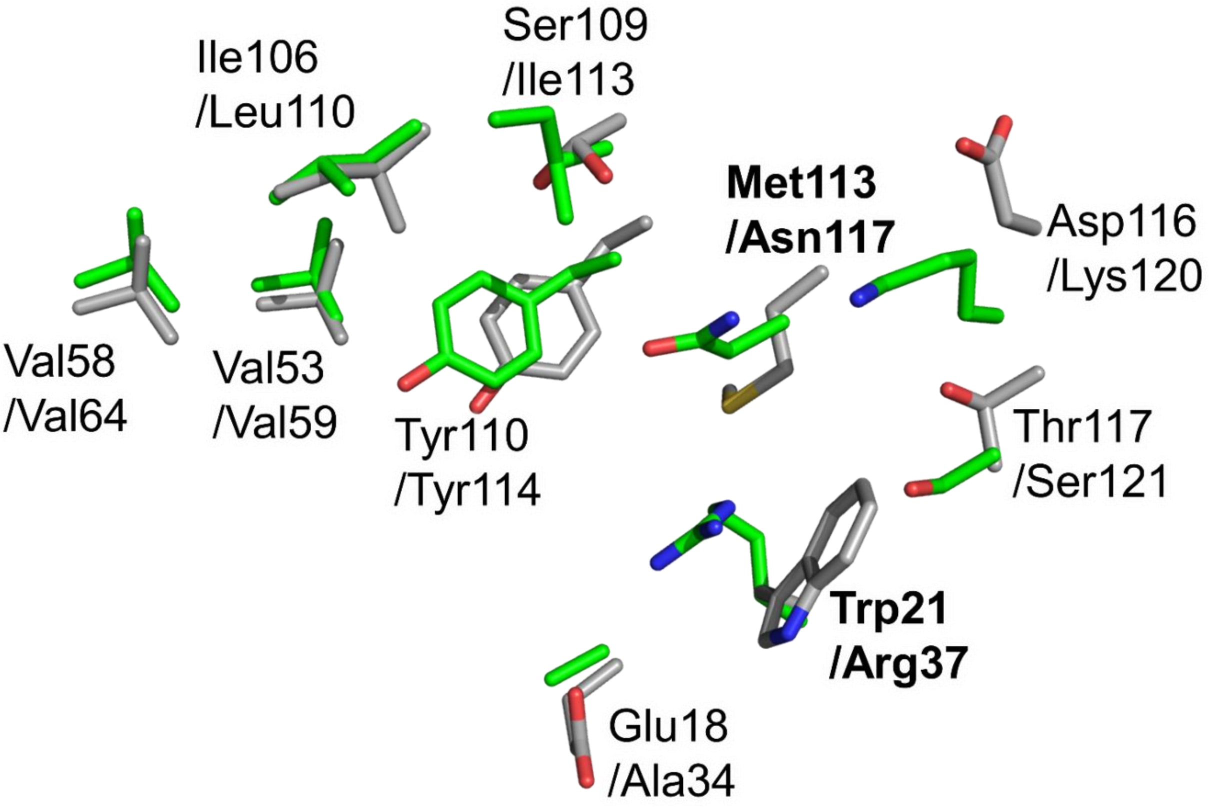

Supplement: S3 Fig — (PDF) [file pntd.0008339.s005.pdf]
