# Supplementary material for: Spiro-containing derivatives show antiparasitic activity against Trypanosoma brucei through inhibition of the trypanothione reductase enzyme
Source: PLoS Negl Trop Dis. 2020 May 21;14(5):e0008339. doi: 10.1371/journal.pntd.0008339 (PMC7269337; doi:10.1371/journal.pntd.0008339)

Supporting Information

S4 Fig. The hydrophobic and positively charged moieties of compound **1** appear to be complementary to the *Tb*TR cavity surface. site 1 (A) or site 2 (B).

A

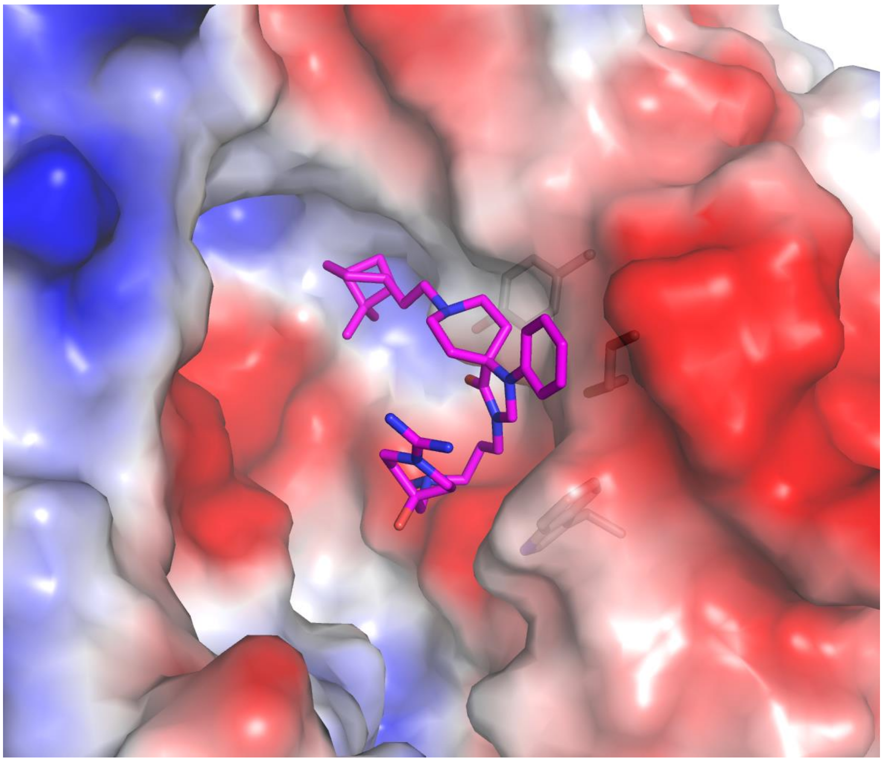

B

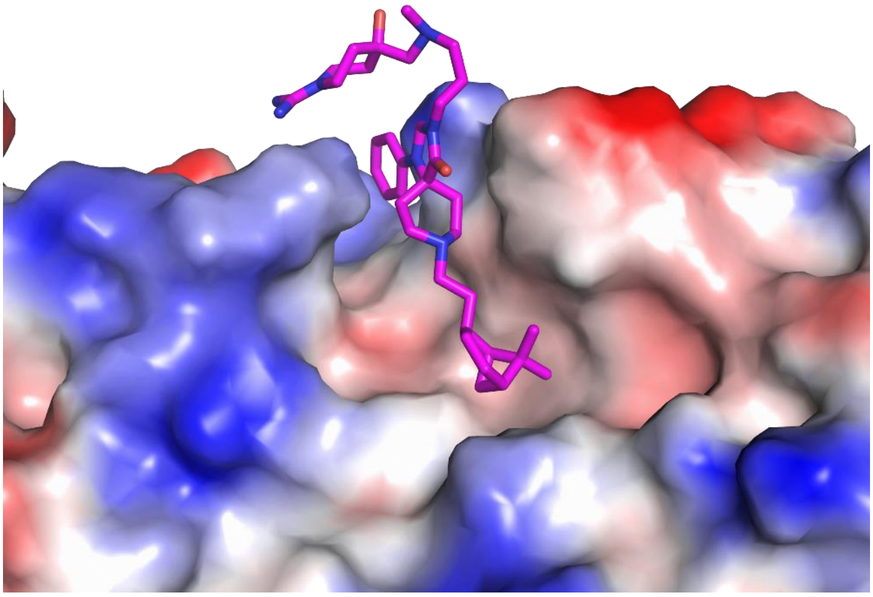

Supplement: S4 Fig — site 1 (A) or site 2 (B). (PDF) [file pntd.0008339.s006.pdf]
