# Supplementary material for: Spiro-containing derivatives show antiparasitic activity against Trypanosoma brucei through inhibition of the trypanothione reductase enzyme
Source: PLoS Negl Trop Dis. 2020 May 21;14(5):e0008339. doi: 10.1371/journal.pntd.0008339 (PMC7269337; doi:10.1371/journal.pntd.0008339)
Supplement: S5 Fig — Each experimental point is the average of three replicates. (PDF) [file pntd.0008339.s007.pdf]

Supporting Information

S5 Fig. inhibition of *Leismania infantum* trypanothione reductase (*Li*TR) by Compound **1.** Each experimental point is the average of three replicates.

The following method was used to determine the inhibition of the *Li*TR activity:
L. infantum TR was cloned and purified as previously described.[1] Bovine serum albumin (BSA), NADPH, auranofin, hGR, oxidized glutathione (GSSG) and DTNB were purchased from Sigma-Aldrich (St. Louis, MO); oxidized trypanothione (TS_2) was purchased from Bachem (Bubendorf, Switzerland); the NADPH-Glo kit was purchased from Promega (Madison, WI, U.S.A.). Compounds, dissolved in DMSO, were transferred to 384-well white plates (Greiner Bio One, Frickenhausen, Germany) using the acoustic droplet ejection technology (ATS-100, EDC Biosystems, U.S.A.) to reach the desired final concentration. The TR enzymatic reaction was performed by addition of 0.1 nM TR, 12.5 µM NAPDH, 15 µM TS_2 in 50 mM HEPES (pH 7.4), 40 mM NaCl, 0.01% BSA in a final volume of 15 µL. After 60 min of incubation at room temperature, an equal volume of NADPH-Glo reagent was added and the luminescent signal was acquired by an EnVision plate reader (PerkinElmer, Waltham, MA, U.S.A.). In fact the NADPH-Glo signal was demonstrated to stabilize after 30 minute (Figure S1) according to the reagent data sheet. In addition, in order to make sure that the production of a reducing agent (i.e. $T(SH)_2$) by the TR activity was not going to interfere with the assay, and in absence of $T(SH)_2$ as purified reagent, the effect of DTT and GSH was tested on the NADPH-Glo reaction resulting in no interference at concentrations relevant for the current setup (Figure S2). The hGR assay was performed by addition of 0.5 nM hGR, 10 µM GSSG, 20 µM NADPH in 50 mM HEPES (pH 7.4), 40 mM NaCl, 0.01% BSA in a final volume of 15 µL. After 30 min incubation at room temperature 15 µl of NADPH-Glo kit was added to reveal the signal. The 5,5′-dithiobis(2-nitrobenzoic acid) (DTNB) assay was performed using 2 nM TR, 100 µM NADPH, 4 µM TS_2 and 200 µM DTNB in 40 mM HEPES (pH 7.4), 1 mM EDTA, 0.01% BSA and 0.05% tween-20 in a final volume of 50 µl. The absorbance signal (412 nm) was acquired 15 minutes post incubation at room temperature using the plate reader (Safire2, Tecan, Switzerland). Results were analyzed using Prism software (GraphPad, San Diego, CA, U.S.A.) and Vortex (Dotmatics, Bioshops Stortford, UK). Dose-response curves were fitted by four-parameter logistic regression.

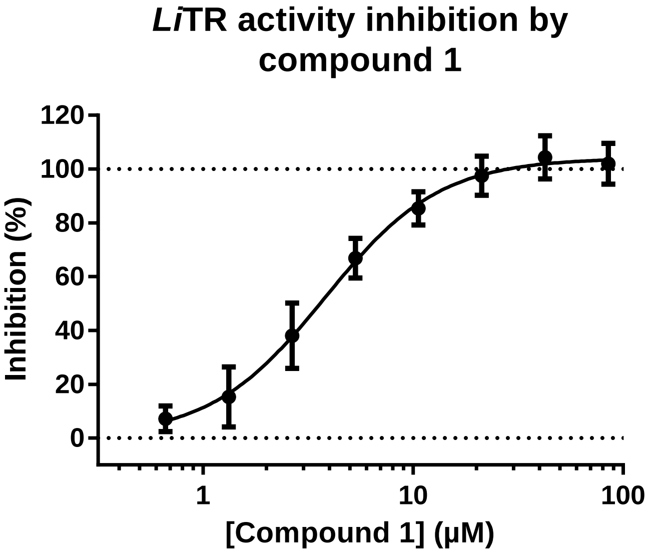

IC_{50} = 3.8 µM ± 0.6 (n=3)

References

[1] Baiocco P, Colotti G, Franceschini S, Ilari A. Molecular basis of antimony treatment in leishmaniasis. Journal of medicinal chemistry. 2009;52(8):2603-12. doi: 10.1021/jm900185q. PubMed PMID: 19317451
