# Supplementary material for: Spiro-containing derivatives show antiparasitic activity against Trypanosoma brucei through inhibition of the trypanothione reductase enzyme
Source: PLoS Negl Trop Dis. 2020 May 21;14(5):e0008339. doi: 10.1371/journal.pntd.0008339 (PMC7269337; doi:10.1371/journal.pntd.0008339)
Supplement: Attachment — Submitted filename: Turcano 2019 Response to reviewers.docx [file pntd.0008339.s008.docx]

Dear Grace Adira Murilla

Associate editor

PLOS Neglected Tropical Diseases

Thank you for the opportunity to submit a new version of our manuscript.

We want to thank the reviewers for their valid comments which have, hopefully, improved the quality of our manuscript. We aimed at replying each comment as clearly as possible and included the revisions in the manuscript wherever we thought that it was needed.

A version of the revised manuscript has also been uploaded. Changes are highlighted with the “Track Changes” option of Microsoft Word in the “marked” version of the manuscript.

Below, we reported how we have addressed each of the reviewer’s comments. The reviewer’s comments are reported in black whereas the answers are reported in blue. Please note that the indicated lines refer to the “marked document”

Reviewer #1

Minor revisions/suggestions:

Lines 94/5 – suggested alternative wording: ‘The X-ray structure of compound 1-bound TbTR revealed that compound 1 binds a TR-specific hydrophobic pocket in the TS2 binding site.’

Lines 101 - The sentence was rephrased as: ‘The X-ray structure of the *Tb*TR/compound **1** complex allowed the identification of a new pocket in the TS_2_ binding site where compound **1** binds’

Line 138/9 – suggested alternative wording: ‘Human glutathione reductase (hGR) activity was assayed as described by Turcano et al [21]’

Lines 157 - The sentence was rephrased as: ‘The human glutathione reductase (hGR) activity assay was carried out as described by Turcano et al.[22].’

Line 347 – complementarity

Corrected

Line 394 – replace ‘added’ with ‘supplemented’

Corrected

Micromolar IC50 values are reported for compound 1, however the x-axes in Fig 7 are labelled nM.

Thank you for having spot this typo. The figure is now corrected.

Line 396 – suggested alternative wording: ‘Fig. 7A shows that compound 1 inhibits TbTR activity in a dose-dependent manner…’

Lines 493 - The sentence was rephrased as: ‘**Fig. 7A** shows that compound **1** is active in a dose-response manner’

Line 402 – consistent use of italics and abbreviated species format (T. brucei)

Consistency was verified

Reviewer #2

Minor revisions/suggestions:

There are some typographical errors

We are sorry for this inconvenience, the manuscript was revised and scanned for typos.

Reviewer #1

Summary and general comments

Turcano and colleagues describe a new lead compound with activity against trypanothione reductase (TR) and in vitro cultured Trypanosoma brucei at low micromolar concentrations. They define its binding to TR, demonstrate its in vitro selectivity for TR versus human glutathione reductase (hGR), and present data indicating enzyme inhibition via an interaction with the trypanothione binding site. The results are interesting and clearly presented. I only have a couple of minor comments.

We would like to thank the reviewer for her/his comment.

The authors discuss the toxicity of the current anti-trypanosomal drugs (highlighting pentamidine and melarsoprol), the need for new treatment development (lines 70-4) and the identification of new biochemical pathways for targeting (lines 74-6). They should also mention recent successes in drug development, including the oxaboroles and the approval of fexnidazole.

Thank you for pointing this out. A sentence covering this notion is now added (lines 72)

The available drugs are still limited and other challenges remain, such as the need for drugs to cross the blood brain barrier in late stage HAT, which aren’t mentioned in the introduction – interestingly, the brain penetrance of other spiro core-containing compounds is highlighted in the discussion (lines 441-3).

This is now also mentioned in the introduction sections lines 74-75

The authors describe the conservation of TR within the kinetoplastid parasites and suggest their lead compound may be applicable to Leishmania and T. cruzi (lines 432-4). Although not essential, this conclusion could be strengthened by the inclusion of data on compound activity against Leishmania promastigotes (and recombinant LiTR?), as per the authors’ 2018 paper (PMID 30475811).

We indeed have these data that is pretty in agreement with the hypothesis. For this reason we accepted the suggestion and added fig. S-7 and a mention to the result in lines 414-417

Reviewer #2

Summary and general comments

No comments and suggestions
