# Supplementary material for: Spiro-containing derivatives show antiparasitic activity against Trypanosoma brucei through inhibition of the trypanothione reductase enzyme
Source: PLoS Negl Trop Dis. 2020 May 21;14(5):e0008339. doi: 10.1371/journal.pntd.0008339 (PMC7269337; doi:10.1371/journal.pntd.0008339)
Supplement: Attachment — Submitted filename: Turcano 2020 Response to reviewers.docx [file pntd.0008339.s009.docx]

Dear Grace Adira Murilla

Associate editor

PLOS Neglected Tropical Diseases

Thank you for the opportunity to submit a new version of our manuscript.

We want to thank the reviewers for their valid comments which have, hopefully, improved the quality of our manuscript. We aimed at replying each comment as clearly as possible and included the revisions in the manuscript wherever we thought that it was needed.

A version of the revised manuscript has also been uploaded. Changes are highlighted with the “Track Changes” option of Microsoft Word in the “marked” version of the manuscript.

Below, we reported how we have addressed each of the reviewer’s comments. The reviewer’s comments are reported in black whereas the answers are reported in blue. Please note that the indicated lines refer to the “marked document”

Reviewer #1

‘Lines 72-73: More recently, oral compounds like fexinidazole or oxaboroles have come to fruition’.
Fexinidazole is the first all-oral treatment for both early and late stages of Trypanosoma brucei gambiense sleeping sickness; distribution in endemic countries to start in 2019.
This is significant achievement in the treatment of sleeping sickness, and should be included in the response

We agree with the Reviewer’s point above. In our previous revision we already added a mention to this that has been strengthened in the new version with the addition of a sentence and reference dedicated to fexinidazole. Lines 74-75.

Best regards

Alberto Bresciani
